# Late Cardiological Sequelae and Long-Term Monitoring in Classical Hodgkin Lymphoma and Diffuse Large B-Cell Lymphoma Survivors: A Systematic Review by the Fondazione Italiana Linfomi

**DOI:** 10.3390/cancers14010061

**Published:** 2021-12-23

**Authors:** Stefano Oliva, Agata Puzzovivo, Chiara Gerardi, Eleonora Allocati, Vitaliana De Sanctis, Carla Minoia, Tetiana Skrypets, Attilio Guarini, Guido Gini

**Affiliations:** 1 Cardioncology Unit, IRCCS Istituto Tumori “Giovanni Paolo II”, 70124 Bari, Italy; stefanoliva66@gmail.com; 2 Istituto di Ricerche Farmacologiche “Mario Negri” IRCCS, 20156 Milan, Italy; chiara.gerardi@marionegri.it (C.G.); eleonora.allocati@marionegri.it (E.A.); 3 Department of Medicine and Surgery and Translational Medicine, “Sapienza” University of Rome, Radiotherapy Oncology, St. Andrea Hospital, 00189 Rome, Italy; vitaliana.desanctis@uniroma1.it; 4 Hematology Unit, IRCCS Istituto Tumori “Giovanni Paolo II”, 70124 Bari, Italy; c.minoia@oncologico.bari.it (C.M.); tetianaskrypets@gmail.com (T.S.); attilioguarini@oncologico.bari.it (A.G.); 5 Clinic of Hematology, AOU Ospedali Riuniti Ancona-Università Politecnica delle Marche, 60126 Ancona, Italy; guido.gini@ospedaliriuniti.marche.it

**Keywords:** survivors, classical Hodgkin lymphoma, diffuse large B-cell lymphoma, cardiotoxicity, cardiovascular, echocardiography, monitoring, risk factors, incidence, systematic review

## Abstract

**Simple Summary:**

The multidisciplinary team of Fondazione Italiana Linfomi researchers conducted a systematic review of the literature (PubMed, EMBASE, Cochrane database) regarding incidence, comparison between systemic therapies and radiotherapy (RT) (old versus modern techniques), and the better monitoring of long-term classical Hodgkin lymphoma and diffuse large B-cell lymphoma survivors on late cardiological sequelae. The research focused on patients treated in adulthood and with first- or second-line antineoplastic therapies, including autologous stem cell transplant. Our purpose was to provide an overall and updated picture of the incidence of the phenomenon, the risk factors, and the updated early detection and follow-up strategies.

**Abstract:**

Cardiotoxicity represents the most frequent cause with higher morbidity and mortality among long-term sequelae affecting classical Hodgkin lymphoma (cHL) and diffuse large B-cell lymphoma (DLBCL) patients. The multidisciplinary team of Fondazione Italiana Linfomi (FIL) researchers, with the methodological guide of Istituto di Ricerche Farmacologiche “Mario Negri”, conducted a systematic review of the literature (PubMed, EMBASE, Cochrane database) according to the Preferred Reporting Items for Systematic Reviews and Meta-Analyses (PRISMA) guidelines, in order to analyze the following aspects of cHL and DLBCL survivorship: (i) incidence of cardiovascular disease (CVD); (ii) risk of long-term CVD with the use of less cardiotoxic therapies (reduced-field radiotherapy and liposomal doxorubicin); and (iii) preferable cardiovascular monitoring for left ventricular (LV) dysfunction, coronary heart disease (CHD) and valvular disease (VHD). After the screening of 659 abstracts and related 113 full-text papers, 23 publications were eligible for data extraction and included in the final sample. There was an increased risk for CVD in cHL survivors of 3.6 for myocardial infarction and 4.9 for congestive heart failure (CHF) in comparison to the general population; the risk increased over the years of follow-up. In addition, DLBCL patients presented a 29% increased risk for CHF. New radiotherapy techniques suggested reduced risk of late CVD, but only dosimetric studies were available. The optimal monitoring of LV function by 2D-STE echocardiography should be structured according to individual CV risk, mainly considering as risk factors a cumulative doxorubicine dose >250 mg per square meter (m^2^) and mediastinal radiotherapy >30 Gy, age at treatment <25 years and age at evaluation >60 years, evaluating LV ejection fraction, global longitudinal strain, and global circumferential strain. The evaluation for asymptomatic CHD should be offered starting from the 10th year after mediastinal RT, considering ECG, stress echo, or coronary artery calcium (CAC) score. Given the suggested increased risks of cardiovascular outcomes in lymphoma survivors compared to the general population, tailored screening and prevention programs may be warranted to offset the future burden of disease.

## 1. Introduction

Cardiotoxicity is the most frequent cause of morbidity and mortality occurring in lymphoma survivors after second cancers [1,2,3]. Numerous studies on the long-term follow-up of classical Hodgkin lymphoma (cHL) patients have shown that cardiovascular disease (CVD) also can occur more than 20 years after treatment. CVD is defined as coronary heart disease (CHD), which included myocardial infarction (MI), percutaneous coronary intervention, coronary artery bypass graft surgery, or >75% stenosis on coronary angiogram or autopsy, cardiomyopathy, valvular heart disease (VHD), or pericardial disease. Patients treated at a young age may therefore present severe comorbidities that affect their quality of life and overall survival [1]. Causes of cardiovascular long-term toxicity are well recognized in dose-dependent, anthracycline-including chemotherapy and mediastinal radiotherapy. However, some individual risk factors, such as family history of CVD, also play a key role. Hypertension, high cholesterol level, diabetes, and smoking also contribute to the onset of CVD in the general population [4,5]. A sedentary lifestyle and obesity could also increase the risk of developing CVD in lymphoma survivors [6].

There is a variation in the definition of cancer-therapy-related cardiac dysfunction across guidelines, position statements, and oncology trials. The risk of CVD in patients treated for cHL appears to increase over time, never reaching a plateau in the incidence curve [7], and it results to be 2 to 5 times higher than in the general aged-matched population [8]. Even in patients treated for non-Hodgkin’s lymphoma (NHL) the cumulative risk of CVD increases over time and is higher compared to the general population [9]. Advanced age and being overweight seem to represent additional risk factors in this population [10,11,12]. Patients undergoing a salvage chemotherapy and autologous hematopoietic stem cell transplantation (ASCT) could be considered as a population with a greater risk of CVD than the general population [13]. The risk of CHF after ASCT increases 4.5-fold compared with the age- and sex-matched general population, with an absolute excess risk (AER) of 0.96% per year [14]. The AER is defined as the mean excess number of CHF cases per 10,000 survivors per year.

Despite these aspects, constant monitoring in asymptomatic patients for CVD is not currently recommended. The USA guidelines focus on a strict control of cardiovascular risk factors, while in cHL survivors an echocardiographic examination is suggested at 10-year intervals, starting from the 10th year of remission [15]. For patients who have received anthracyclines, the European guidelines recommend cardiovascular follow-up starting after 2 years of remission and then periodically, without a defined timing. For patients who have undergone mediastinal radiotherapy, an evaluation of CHD and valvular pathology is indicated from the 5th year of remission, and then every 3–5 years [16]. It is important to highlight that these guidelines are not designed for lymphoma survivors. In recent years, numerous approaches have been developed with the aim of reducing the risk of anthracycline-related cardiotoxicity, both with the use of cardioprotective drugs [17,18,19] and liposomal anthracyclines [20,21]. Additionally, in the last decades we have observed a reduction of volumes and radiation doses, as well as an improvement of radiotherapy techniques, due to the introduction of a more conformational approach such as intensity-modulated radiation therapy (IMRT) [22,23] and the use of deep inspiration breath-hold radiotherapy and proton therapy [24]. Recently, clinical research has focused on the assessment of cardiovascular damage through different study techniques: new echocardiographic measurements and echocardiographic parameters such as global longitudinal strain (GLS) [25]. Cardiac magnetic resonance is recommended if the quality of the echocardiogram is suboptimal [26]. Moreover, promising results have been obtained with the evaluation of some serological markers, primarily troponin I [27]. An increase of troponin I in the serum of patients treated with anthracyclines could identify a cohort at greater risk of future cardiotoxic events, who would benefit from treatment with angiotensin-converting enzyme inhibitors (ACEi) as a secondary prevention [28]. Despite available promising studies, the heterogeneity of cardiotoxic events and the absence of large multicenter RCTs have not allowed us to obtain reliable and validated individual risk scores.

Taking into account previously mentioned data, the Fondazione Italiana Linfomi conducted a systematic review with the aim of evaluating specific aspects of long-term CVD in the selected population of cHL and diffuse large B-cell lymphoma (DLBCL) survivors, in remission for more than 5 years and treated both with first-line therapy and salvage chemotherapy and ASCT. Specifically, the systematic review focused on: (I) the evaluation of the incidence of CVD in cHL and DLBCL survivors; (II) the assessment of the incidence of CVD with the use of less cardiotoxic therapies: reduced-field/dose of radiotherapy and liposomal doxorubicin; and (III) the definition of the preferable cardiovascular monitoring of the subset of long-term cHL and DLBCL survivors.

## 2. Materials and Methods

This systematic review is one publication of a sequence of different reviews assessing the management and follow up of long-term lymphoma survivors, and upholding the Fondazione Italiana Linfoma (FIL) position paper. The aim of this project, as well as the clinical questions and PICOs for each question, were discussed and accepted by the FIL long-term survivor commission and presented at the FIL congress in 2019. We utilized the Preferred Reporting Items for Systematic Reviews and Meta-Analyses (PRISMA) guidelines to record the results [29].

### 2.1. Study Identification

MEDLINE (via PubMed), the Cochrane Library, and EMBASE were systematically researched from January 1990 to September 2020 without restrictions on the language or type of publication. Search terms included extensive controlled vocabulary (MeSH and EMTREE) and free-text keywords, combining the conditions (HL DLBCL), interventions (e.g., radiotherapies and chemotherapies), and outcomes of interest (e.g., cardiac mortality, cardiotoxicity, cardiovascular toxicity). The Supplementary Materials (Table S1) contain details on the search strategies. We examined the reference lists of significant studies to recall further congress abstracts and studies, and searched study registries for studies that are not yet published or ongoing.

### 2.2. Eligibility Criteria

We incorporated both primary studies (prospective and retrospective cohort studies, randomized controlled trials, and registry studies) and systematic reviews, including these study designs. We included studies involving cHL or DLBCL long-term survivors (≥5 years disease- or treatment-free), and adults with an age ≥18 years at diagnosis. Included studies assessed: (I) the incidence of CVD in HL and DLBCL survivors; (II) the clinical impact of the use of less cardiotoxic therapies: reduced-field radiotherapy and liposomal doxorubicin; and (III) the preferable cardiovascular monitoring of this subset of patients.

Table 1 shows the clinical questions and corresponding PICO considered in this review.

The evaluation concentrated on patients treated with first- or second-line therapy including an ASCT. Patients undergoing allogeneic stem cell transplantation were excluded.

### 2.3. Risk of Bias and Quality of Evidence Assessment

The AMSTAR 2 tool [30] was used to evaluate the methodological quality of the systematic reviews, the Cochrane Risk of Bias (ROB) [31] to determine the risk of bias for the RCTs, and the New Castle Ottawa Scale to assess the quality of cohort and registry studies [32]. The risk of bias and quality of evidence assessment was performed by one reviewer and confirmed by a second one.

### 2.4. Study Selection and Data Extraction

One reviewer screened the title and abstract to identify the studies, and evaluated the full-text publication to validate the eligibility and extract the meaningful data from the selected trials. A second reviewer verified the eligibility and the data extraction to improve the accuracy of the process. A third author arbitrarily and by consent resolved any discrepancies.

Data extrapolated from each study included the following predefined elements: study ID (first author, year of publication); reference; other publication; study design; population; study duration; follow-up; sample size; intervention; control group; outcome measure; main results; conclusion; and risk of bias/quality assessment. A predefined spreadsheet (Microsoft Corporation^®^,Redmond, WA, USA) was used for data extraction and is included in the Supplementary Materials, as is the full assessment of risk of bias.

### 2.5. Data Synthesis

In consideration of an expected heterogeneity between the included studies, we did not pool data in meta-analyses. For each clinical question, the selected studies providing meaningful data were summarized narratively and listed to underline differences and similarities in methods and results.

## 3. Results

### 3.1. Incidence of CVD in cHL and DLBCL Survivors

What is the incidence of CVD in long-term survivors of cHL or DLBCL after the first- or second-line treatment (including ASCT)?

**Figure 1:**
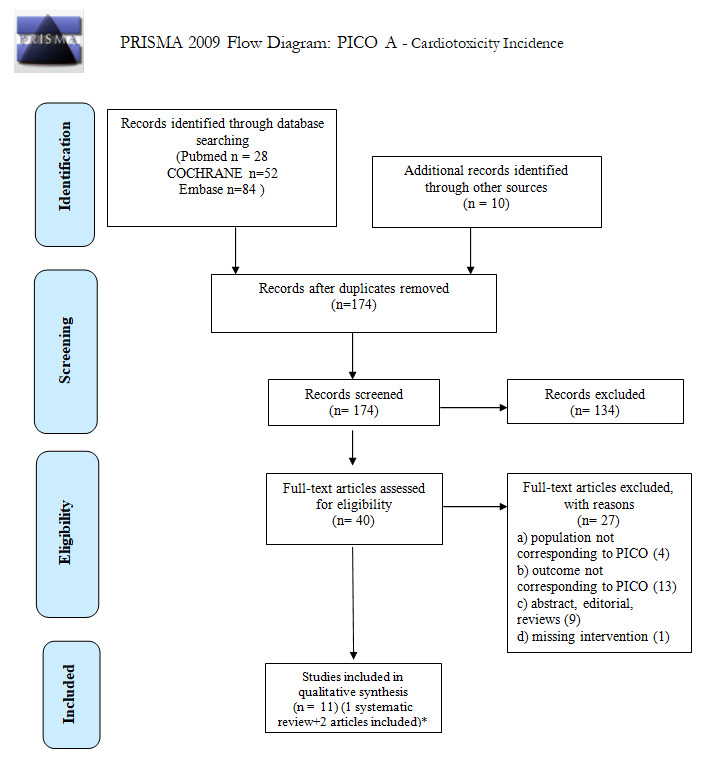
PRISMA 2009 Flow diagram for PICO A: cardiotoxicity incidence. * Of 13 included studies, 2 studies [7,8] were included in Stone’s systematic review [33] and were not evaluated separately.

We screened 174 abstracts, and 40 relevant publications were retrieved as full text. Of these, 27 studies were excluded and 13 were included in the final sample and relative analysis. The main exclusion criteria were: the presence of a pooled population including non-Hodgkin’s lymphomas whose specific histotype had been not detailed in the manuscripts, not allowing possible conclusions on DLBCL; a population not matching the definition of long-term survivors; and the lack of an intervention impacting on outcome. Figure 1 (PRISMA flowchart) shows details of the entire screening process, including reasons for full-text exclusion. Details on full-text-eligibility data extraction and assessment of risk of bias are reported in Tables S2 and S3, respectively.

The results are summarized in Table 2.

Of 13 included studies, 2 studies [7,8] were included in the Stone’s systematic review [33] and not evaluated separately. These studies were conducted from 1964 to 2018. Most publications focused on HL survivors.

Four publications were focused on the incidence of cardiotoxicity in patients treated for HL after first-line treatment [34,35,40,42]; one publication was focused on the incidence and predictors of congestive heart failure after ASCT for HL and NHL [39]. One paper analyzed doxorubicin, cardiac risk factors, and cardiotoxicity in DLBCL patients [58]. The systematic review of Stone et al. identified 22 studies (32,438 HL and NHL survivors).

The systematic review of Stone et al. was conducted using EMBASE, MEDLINE, and CINAHL databases, from the initiation of the different databases to November 2016, with additional searches completed through June 2018 [33]. Stone et al. included observational studies assessing CVD incidence in HL and NHL survivors for at least 5 years from the initial diagnosis, or if the study had a median follow-up of 10 years. The authors identified 7729 records; of these, they included only 22 full texts. Subgroup analyses were performed to detect the incidence of specific CVD (myocardial disease, pericardial disease, VHD, coronary heart disease, arrhythmia, and cerebrovascular disease). The included studies had an average median duration of follow-up of 14.7 years (range: 8.4 to 23.3; IQR: 13.6 to 18.0), median age at diagnosis was 27.1 years, and the median percentage of females was 46.4%. This systematic review highlighted the elevated risk of developing CVD in lymphoma survivors compared to the general population. Lymphoma survivors had statistically significant 2- to 3-fold increases in risk for almost all subtypes of CVD. Lymphoma survivors appeared to be at risk primarily of pericardial disease (HL: 10.67, 95% confidence interval (CI), 7.75–14.69) and valve disease (HL: 13.10, 95% Cl, 7.41–23.16). The risk of bias included studies was in general low; all works had a study population of lymphoma survivors and a control group drawn from the same population. One of the limitations of this work is the heterogeneity of patient treatments (studies on patients undergoing older treatments were also included). In recent years, therapeutic approaches have changed and improved, and it is therefore possible that in the authors’ analyses, there was an overestimation of the effects that actually occur in current practice. Another limitation of the systematic review is that the authors considered studies with a nonhomogeneous population (NHL and HL survivors with different histotypes). Despite this, the overall weight of associations was strong enough to encourage the use of specific cardiovascular screening, prevention, and surveillance programs in order to counteract the future burden of CVD within this population of lymphoma survivors.

Holtzman A et al. reviewed, in their retrospective cohort study, the medical records of 365 HL patients who underwent primary RT between 1965 and 1995 [34]. The authors’ aim was to evaluate the long-term outcomes of patients with at least 20 years of minimum potential follow-up time after RT. The radiotherapy techniques have been different over the years (Co gamma ray teletherapy two-dimensional planning and delivery techniques until 1978; after linear accelerators until 19t5; three-dimensional imaging was introduced in 1984). A total of 11 patients received involved-field RT (IFRT), 25 received extended-field RT (EFRT), 149 subtotal irradiation (STNI), and 146 total nodal irradiation (TNI). A total of 160 patients were treated on RT alone; 205 also received adjuvant chemotherapy (3–6 cycles of mustargen, oncovin, procarbazine, and prednisone or adriamycin, bleomycin, vinblastine, and dacarbazine) or salvage chemotherapy after RT.

The incidence and prevalence of cardiovascular disease increased over time, with 37 events in 33 patients (9%) at 10 years, 70 events in 56 patients (15%) at 20 years, 132 events in 95 patients (26%) at 30 years, and 157 events in 109 patients (30%) at 40 years of follow-up. Additionally, 47% of 91 patients with >30 years of follow-up and 74% of 23 patients with >40 years of follow-up had at least one cardiovascular event. RT and chemotherapy lowered the mean age of onset of cardiac events. The data from the study underscored the importance of reducing integral dose to nontargeted tissues in HL patients receiving RT. The study quality was intermediate. The main strength of these works was the length of follow-up (median effective follow-up of 27 years in survivors).

Maraldo et al. tried in their study to quantify the effect of anthracyclines, vinca-alkaloids, and specific radiation dose levels of RT on cardiovascular disease risk in order to facilitate the definition of patients’ personal risk of cardiotoxicity [35]. In 2009–10, a Life Situation Questionnaire (LSQ) was sent to patients by mail to evaluate late-onset consequences of cHL treatment in patients who were included in nine randomized trials of the European Organization for Research and Treatment of Cancer (EORTC) and of the Group d’Etude del Lymphomes de l’Adult (GELA) between 1964 and 2004. The trials are identified with the following codes: H1, H2, H5, H3b4, H6, H7, H8, H34, and H9 [36]. Over the years, radiation therapy and chemotherapy treatments were modified. Patients in the H1, H2, and H5 trials were previously treated with radiation therapy alone, whereas patients in the H3b4, H6, H7, H8, H34, and H9 trials were more likely to receive combined modality treatment with RT, anthracyclines, and vinca-alkaloids. A total of 1919 patients of 115 LSQ-participating centers answered (median age at treatment 30 years; median follow-up duration 9 years); 1238 cardiovascular events occurred in 703 patients, most were ischemic heart disease (19%), arrhythmia (16%), CHF (12%), and VHD (11%). The mean heart radiation dose per 1 Gy increase and the dose of anthracyclines per 50 mg/m^2^ in cumulative dose were significant predictors of CVD (HR 1.01 (95% CI 1.006–1.024), *p* = 0.0014; HR 1.08 (95% CI 1.021–1.137), *p* = 0.0064, respectively). The quality of the study was intermediate, mainly due to selection bias for cases. The main concerns regarded the incomplete outcome data (attrition bias) and the self-reported outcome (reporting bias).

Aleman et al. assessed the long-term risk of specific CVD in a population of 1474 HL patients treated between 1965 and 1995 who survived for at least 5 years. A strength of the study was the long and almost full follow-up and the accessibility of comprehensive data on the treatment carried out (radiation fields and chemotherapy drugs) [42]. A total of 28% of the entire study population received RT only, 38% received RT and chemotherapy without anthracyclines, and 29% underwent RT and chemotherapy including anthracyclines. At least 5 years after the diagnosis of HL, 619 cardiovascular events occurred in 354 of the 1474 patients. The most frequently detected cardiovascular diseases were valvular disorders (160), angina pectoris (134), and MI (102). The standardized incidence ratios (SIRs) of the risk of MI and congestive heart failure were 3.6 and 4.9, respectively, resulting in 25.6 cases of congestive heart failure and 35.7 cases of myocardial infarction per 10,000 patients/year compared to the general population. RT increased the risk of angina pectoris, MI, VHD, and CHF 2- to 7-fold. Anthracyclines increased the elevated risks of CHF and VHD from mediastinal radiotherapy (HRs 2.81 and 2.10); the 25-year cumulative incidence of CHF was 7.9% after RT and anthracycline-containing chemotherapy. HL survivors compared to the general population had a 3- to 5-fold increased risk of CVDs, especially with longer follow-up, and there was an increase in absolute excess risk over time. In this study, the case definition was adequate, representativeness of the cases were not adequate, and they included only younger patients (survivors of HL younger than 41 years at treatment).

Hershman et al. set up an observational study to assess the associations of doxorubicin with clinical, demographic, and cardiac variables among adult patients (aged ≥65 years) diagnosed with DLBCL from 1991 to 2002 in the Surveillance, Epidemiology, and End Results (SEER)–Medicare database [58]. In the 8 years after diagnosis, the adjusted CHF-free survival rate was 74% in doxorubicin-treated patients versus 79% in patients not treated with doxorubicin. Any doxorubicin use was associated with a 29% increase in risk of CHF (95% CI, 1.02 to 1.62); CHF risk increased with the number of doxorubicin prescriptions, older age, previous heart disease hypertension, diabetes, and comorbidities. The authors demonstrated that only hypertension had a synergic effect with doxorubicin in the increased the risk of CHF (HR = 1.8; *p* < 0.01). In this study, the case definition, representativeness of the cases, and selection of controls were adequate. 

Clavert et al. analyzed in their retrospective cohort study the late complications in 110 adult patients who underwent reduced-intensity conditioning allogenic stem cell transplantation. The authors described 47% of CVD incidence at 10 years (95% CI 35–59), and main presentations were heart failure (14%) and arterial hypertension (7%). The study lacked data with reference to specific haematological diagnosis and NHL histotypes [36]. 

Baech et al. evaluated the role of the cumulative effect of anthracycline doses in a retrospective cohort study based on the Danish National Lymphoma Registry of 2440 lymphoma patients undergoing first-line immunochemotherapy from 2000–2012 (1.852 DLBCL). Of those patients, 1994 underwent a chemotherapy regimen containing anthracyclines (R-CHOP or R-CHOEP). The control group included patients not treated with anthracyclines. Patients treated with anthracyclines were predominantly diagnosed with DLBCL (88·0%). HR for CHF in patients treated with 3–5 cycles of R-CHOP/CHOEP was 5.0 (95% CI 1.4; 18.5), 6.8 (95% CI 2.0; 23.3) for those treated with 6 cycles, and 13.4 (95% CI 4.0; 45.0) for those who received >6 cycles. For CVDs in general, patients treated with 3–5 cycles of R-CHOP/CHOEP had risks at 1, 5 and 8 years of 2.5%, 10.5%, and 17.2%, respectively. The risks for patients treated with 6 cycles were 2.1%, 9.3%, and 21.2%, and, for patients treated with >6 cycles, they were 5.9%, 15.5%, and 21.7%. The risk estimates for patients treated without anthracyclines were 1.7%, 6.3%, and 7.8% [43]. The quality of the study was intermediate; the sample of exposed patients to anthracyclines contained prevalently DLBCL patients who had been treated homogeneously, the control group of unexposed patients presented prevalently a different lymphoma histotype (follicular lymphoma), and not all patients reached the adequate follow-up (median 38 months).

Cutter et al. performed a case-control study focused on the risk for VHD in a cohort of 1852 5-year HL survivors treated between 1965 and 1995 [41]. A total of 89 case patients with VHD (at least moderate severity) were identified (66 severe), and 200 controls. Aortic and mitral valves (63 and 42, respectively) were most frequently involved. The authors made quantitative estimates of the relationship between radiation dose and the risk of at least moderate VHD. For radiation doses of 0, below or equal to 30, between 31–35, between 36–40, and above 40 Gy, the approximate cumulative risks at 30 years were 1.6%, 3.0%, 6.4%, 9.3%, and 12.4%, respectively; therefore, the authors demonstrated that for doses above 30 Gy, the percentage growth in VHD rate per Gy rose gradually with increasing dose. The splenectomy increased the VHD rate by a factor of 2.3 (*p* = 0.02). The quality of this study according to the Newcastle–Ottawa Scale (NOS) was high, the case definition was adequate, and the selection and exposure of control patients was good (controls were matched for lymphoma diagnosis date, gender, and age). The authors extrapolated treatment and follow-up data from medical records.

Matasar MJ et al. determined late morbidity for 746 adults treated with a first-line therapy for HL at a single center from 1975 to 2000 [40]. All six studies were single-center phase II trials of combined modality therapy for HL: mechlorethamine/vincristine/procarbazine/prednisone (MOPP) +RT; MOPP/doxorubicin/bleomycin/vinblastine/dacarbazine (ABVD) + RT; MOPP/doxorubicin, bleomycin, vinblastine (ABV)/lomustine, doxorubicin, vindesine (CAD) + RT; MOPP/ABVD + RT; MOPP + RT; thiotepa, bleomycin, vinblastine (TBV) + RT; and ABVD + RT, except for one group of one study, which was chemotherapy alone (ABVD). The late morbidity research data were available for 238 patients (45.8%). Cardiovascular morbidity was reported in 130 (54.6%) patients, of whom 35 had a maximum grade of 1, 54 maximum grade 2, 28 maximum grade 3, and 13 maximum grade 4. Concerning the methodological quality of this study, intermediate quality was detected in an incomplete outcome data; indeed, of the 520 survivors, 213 (41%) were lost to follow-up.

Armenian et al., in an observational study, compared the prevalence and the risk of cardiotoxicity in long-term HL and NHL survivors treated with CT including anthracyclines with sex- and age-matched controls (noncancer patients) [39]. The authors also compared the prevalence of cardiac dysfunction in conventionally treated survivors to those who had undergone hematopoietic cell transplantation (HCT). The authors found that the risk of CHF for HCT survivors was increased 4.5-fold compared with the controls, and the absolute excess risk (AER) was 0.96% per year. They reported a significantly increased risk of CHF with increasing age at HCT. At the same time they compared with a general population (age- and sex-matched), showing a higher risk among the younger cohort. The overall assessment of quality, according to the NOS, was intermediate due to an adequate selection of controls, comparability and ascertainment of exposure of cases and controls, and method of evaluation of the exposure. The study lacked data on NHL histotypes [39].

One nested case-control study presented the cardiovascular late toxicity outcome and in a large cohort of 2617 5-year cHL survivors (median age 32.2 years) treated between 1965 and 1995. Follow-up was complete up to October 2013. The study evaluated the risk of CHD as the first cardiovascular event after lymphoma, according to the radiation dose to the heart and type of chemotherapy. An estimate of the in-field irradiated heart volume was calculated via radiation charts and simulation radiographs. Other clinical and lifestyle factors were collected from medical records and from mailed questionnaires completed by the general practitioners (70% of responses). From the whole cohort, 325 survivors reported a CHD as the first event (median interval from lymphoma: 19 years). For each patient with CHD, four controls who had not developed cardiac disease and were matched for sex, age, and date of HL diagnosis were selected (*n* = 1204). Treatments were variable, but controlled for both case and control with multivariate regression analysis. The authors described a 2.5-fold risk of CHD for patients receiving a mean heart dose (MHD) of 20 Gy from mediastinal radiotherapy. Risk of CHD increased linearly with increasing MHD (excess relative risk (ERR)) per Gray, 7.4%; 95% CI, 3.3% to 14.8%) The authors concluded that early management of cardiovascular risk factors and encouragement of physical activity might reduce CHD in cHL survivors [38]. The overall quality of the study, according to the Newcastle–Ottawa Scale (NOS), was high, due to an optimal case definition and representativeness; good selection of controls; comparability and ascertainment of exposure of cases and controls; and the method of evaluation of the exposure.

Van Nimwegen et al. published another case-control study in the cohort of 2617 5-year cHL survivors [37]. The 25–year cumulative risks of heart failure following mean left ventricular doses of 0–15 Gy, 16–20 Gy, and ≥21 Gy were 4.4%, 6.2%, and 13.3%, respectively, in patients treated without anthracycline-containing CT; and 11.2%, 15.9%, and 32.9%, respectively, in patients treated with anthracyclines. The overall quality of the study, according to the Newcastle–Ottawa Scale (NOS), was high, due to an optimal case definition and representativeness; good selection of controls; comparability and ascertainment of exposure of cases and controls; and the method of evaluation of the exposure.

### 3.2. Risk of Long-Term CVD with the Use of Less Cardiotoxic Therapies: Reduced-Field/Dose of Radiotherapy and Liposomal Doxorubicin

Has the incidence of cardiotoxicity in long-surviving patients with cHL or DLBCL changed with the introduction of modern radiotherapy and liposomal doxorubicin?

**Figure 2:**
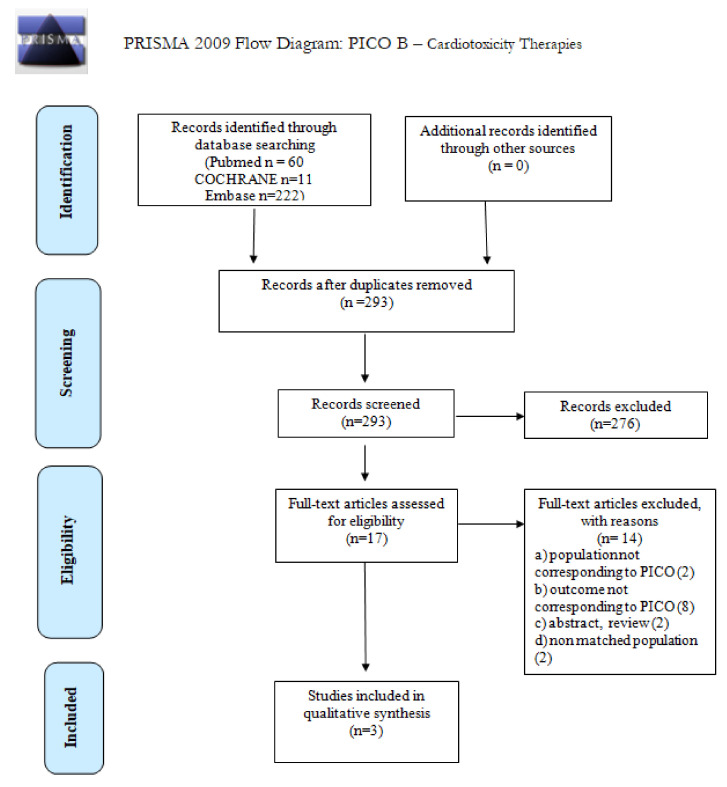
PRISMA 2009 Flow diagram for PICO B: cardiotoxic therapies.

We screened 293 abstracts, and then 17 relevant publications were retrieved as full texts. Of these, 14 studies were excluded and 3 were included in the final sample and relative analysis. Details of the entire screening process, including reasons for full-text exclusion, are reported in Figure 2 (PRISMA flowchart). Details on full-text-eligibility data extraction and assessment of risk of bias are reported in Tables S2 and S3, respectively. The results are summarized in Table 2.

The included studies concerned cHL survivors, and focused mainly on the risk of death for cardiac disease or rates of cardiovascular disease associated with different doses or techniques of radiotherapy. Of the three included studies, two were retrospective, and one was a systematic review.

The first retrospective study was conducted using a multi-institutional database of 1541 early-stage cHL patients treated from 1968 to 2007 with radiotherapy (mediastinal/mantle field); deaths were recorded. According to treatment periods as follows: 1968–1982, 1983–1992, and 1993–2007, the 15-year OS rates were 78%, 85%, and 88%, respectively (*p* = 0.0016). The authors concluded that there was a trend of lower risk of death from cardiac disease in more recently treated patients, although without statistical significance, likely due to the small number of events within each treatment era. Furthermore, when involved versus extended field radiation therapy or different levels of radiation dose (<2000 cGy, 2000 to <3600 cGy, or >3600 cGy) were analyzed, there was a trend toward significance for radiation field extent. So, all-cause mortality risk, including cardiovascular events, was significantly lower in patients treated from 1993 to 2007, likely due to the modern cHL therapeutic approach resulting in a higher cure rate, as well as lower treatment-related toxicity from smaller radiation fields [44].

Another retrospective study was conducted in 746 patients between 1975 and 2000 (238 evaluable for the final analysis) after a median follow-up of 22 years [40]. In this cohort, almost all patients were treated with anthracycline-containing chemotherapy and radiation therapy in doses from 20 to 36 Gy, with the last years involving fields radiotherapy rather than extended fields. The authors showed that patients treated with less than 35 Gy (20–30 Gy) did not have lower rates of cardiovascular disease than those receiving 35 or more Gy.

Finally, Fu et al. performed a systematic review focused on contemporary cHL treatment with particular emphasis on modern radiation approaches, such as volume reduction to the involved and/or nodal field, and the new radiotherapy techniques such as intensity modulation and proton therapy, all with the goal of minimizing the radiation exposure of the cardiac substructures [45]. Eligible articles from 1990 to 2016 were considered, but no studies fulfilled all review criteria. So, the authors concluded that from published data, it was not possible to calculate patient-specific cardiac risk correlated to contemporary treatment (radiotherapy and/or chemotherapy)

The quality of the retrospective study by Patel was intermediate, because although the case definition, assessment of the outcomes, and the length of follow-up was good, the main bias could be represented by the lack of specific volumetric and dosimetric data regarding the radiotherapy treatment delivered and the volume of the cardiac substructures irradiated, and the inclusion of patients with an age lower than 20 years [44]; while for the second [45], it was low due to selection bias (the loss of cases at the accrual) and the detection of generic outcomes (cardiovascular toxicity) performed by a survey and not by a direct evaluation (questionnaire).

According to AMSTAR guidelines, the quality of the review was high for the items considered [29].

Despite the large number of reports on the use of liposomal doxorubicin in combination with cyclophosphamide, vincristine, and prednisone instead of doxorubicin in the treatment of elderly patients with DLBCL affected by cardiac disease, the authors did not find any study comparing the long-term cardiovascular risk of this regimen with the standard therapy.

### 3.3. Preferable Cardiovascular Monitoring for cHL and DLBCL Long-Term Survivors

How to perform an optimal cardio-oncological follow-up in long-term survivors of cHL or DLBCL after first- or second-line treatments (including ASCT)? Which parameters to evaluate and how often?

**Figure 3:**
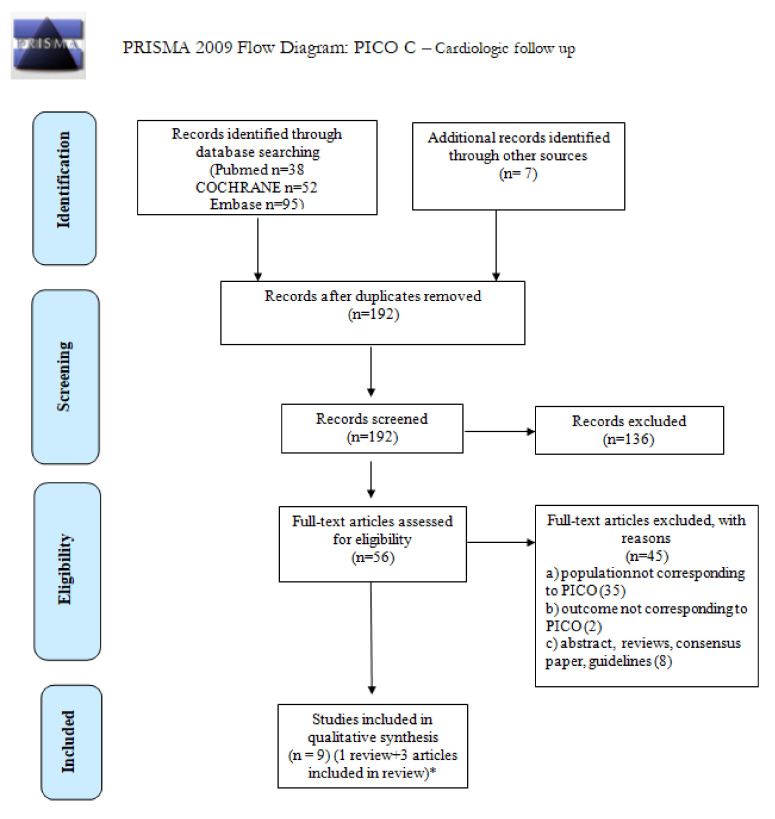
PRISMA 2009 Flow diagram for PICO C: cardiologic follow up. * Of 11 included studies, 3 studies [47,49,50] were included in Nolan’s systematic review [48].

We screened 192 abstracts; 56 relevant publications were retrieved as full text. Seven full texts were added from other sources. A total of nine studies were included in the final sample and relative analysis. Details of the entire screening process, including reasons for full-text exclusion, are reported in Figure 3 (PRISMA flowchart). Details on full-text-eligibility data extraction and assessment of risk of bias are reported in Tables S2 and S3, respectively. The results are summarized in Table 2.

#### 3.3.1. Detection, Monitoring, and Risk Factors for LV Dysfunction

##### Two-Dimensional Echocardiography and Echocardiographic Parameters Other Than LVEF to Early Detect LV Dysfunctions

Echocardiography is currently the method of choice to evaluate serial changes in heart function.

All of the 21 studies (1659 patients) evaluating ventricular function in cHL long-term survivors treated with mediastinal radiotherapy were part of a systematic review by Nolan et al. [48]. Ten used echocardiography as the non-invasive imaging testing modality, eight used radionuclide ventriculography (RNV), two used echocardiography and RNV, and one used cardiac MRI. The authors evaluated: (i) LV systolic dysfunction (19 included studies, 1114 patients, with an average of 11.4 years of follow-up and an average radiation dose of 35.8 Gy); (ii) LV diastolic function (10 included studies, 1009 patients, 12.3 average years of follow-up, average of 36.1 Gy); (iii) right ventricular size and function (5 included studies, 185 patients, followed over an average of 12 years). The authors concluded that LVEF presented limitations for the identification of mild or subclinical LV systolic dysfunction, including significant interobserver variability and reduced sensitivity in early stages of cardiomyopathy. Two-dimensional (2D) speckle tracking strain, as a novel technique, showed more accuracy and reproducibility in diagnosing subclinical systolic dysfunction. LV diastolic function was also evaluated both by echocardiography and by RNV [48]. The risk of bias of this systematic review was intermediate. Data on the only study evaluating cardiac MRI and on multiparametric evaluation by 2D-STE echocardiography will be presented separately [47,49]. In Heidenreich et al., study patients enrolled underwent stress echocardiography and radionuclide perfusion imaging at one stress session [50]; also in this case, data will be presented separately.

In the prospective cross-sectional Norwegian multicenter study performed by Murbreach et al., LVEF confirmed its role in the disclosure of early left ventricular dysfunction in a population of 274 lymphoma survivors treated with doxorubicin and who underwent ASCT [51]. Patients had been treated for cHL or NHL from 1987 to 2008. Conditioning regimens consisted of total-body irradiation (TBI) and high-dose cyclophosphamide from 1987 to 1995. Thereafter, patients received chemotherapy only, including carmustine, etoposide, cytarabine, and melphalan. Patients were divided into four groups: low-dose anthracyclines (<300 mg/m^2^), higher-dose anthracyclines (≥300 mg/m^2^), anthracyclines and low-dose cardiac RT (equivalent to ≤30Gy), and anthracyclines and high-dose cardiac RT (>30 Gy). In univariable regression analyses, a longer observation time since the primary diagnosis, a younger age at lymphoma diagnosis, a cardiac-RT more than 30 Gy, a doxorubicin dose ≥300 mg/m^2^, and three or more lines of chemotherapy before auto hematopoietic cell transplantation were all significantly associated with LV systolic dysfunction, described as LVEF less than 50% or chronic heart failure. In the multivariable analysis, only doxorubicin ≥300 mg/m^2^ and cardiac-RT more than 30 Gy were significantly associated with LV systolic dysfunction, thus establishing independent risk factors for CVD [51]. The quality of the study was optimal/good due to the numerosity of the sample of cases and controls (patients treated with ASCT), the length of the follow-up, the detailed clinical features, and a low interobserved variability.

In patients with the same LVEF (55 ± 8% vs. 56 ± 6%, *p* = 1.0), the global longitudinal strain (GLS) was reduced in those treated with anthracyclines with mediastinal RT for HL compared to the other group receiving mediastinal RT alone or combined RT and regimens without anthracyclines (−16.1 ± 1.9% vs. −17.5 ± 1.7%, respectively). Both patient groups had reduced strain compared to the healthy controls (−20.4 ± 1.7%, both *p* < 0.001). The circumferential strain (GCS) was also reduced in the treatment groups (−18.3 ± 3.2% and −17.8 ± 3.6% vs. −22.5 ± 2.1%, both *p* < 0.001) [48]. The quality of this cohort study was optimal/good.

The importance of a multiparametric evaluation by 2D-STE echocardiography in lymphoma survivors has also been confirmed by other authors. Kang et al. focused on the role of layer-specific strain analysis in assessing the subclinical LV dysfunction in a cohort study including 42 DLBCL survivors (mean age 55.83 ± 17.92 years) treated with epirubicin in comparison to 27 healthy controls (mean age 51.39 ± 13.40 years), while measuring a series of echocardiographic parameters such as GLS, GCS, and radial strain (GRS); and subendocardial, mid-, and subepicardial layer of longitudinal and circumferential strain [46]. The time from last dose of epirubicin to the echocardiographic evaluation was 52.92 ± 22.32 months. The authors demonstrated how conventional parameters of systolic and diastolic function did not show any significant difference between two groups (LVEF 66.04 ± 6.52% for patients and 66.46 ± 5.55% for controls). Multilayer speckle tracking analysis showed significant reduction of the circumferential strain of the subendocardial layer (−37.37 ± 3.79 vs. −32.88 ± 5.23, *p* = 0.000), as well as lower GCS (−27.73% ± 3.37% vs. −24.94% ± 4.14%, *p* = 0.004) and GLS (−21.86 ± 2.38 vs. −20.36 ± 2.58, *p* = 0.016), compared with controls [46]. The quality of the study according to the outcome of our PICO was intermediate, due to the reduced follow-up period for some patients of less than 5 years (even if they were treated in the absence of other cardiotoxic factors such as radiotherapy), while there was good selection of controls, comparability, and ascertainment of exposure of cases and controls, and method of evaluation of the exposure (multiparametric evaluation).

Armenian et al. evaluated 2D-STE echocardiography in a cohort study including 155 survivors (>5 years) of HL (23.9%) and NHL (76.1) (50.3% of patients had received ASCT) between January 1995 and December 2009. The study lacked complete data on NHL histotypes. The prevalence of reduced LVEF, diastolic dysfunction, and abnormal GLS was 8.4%, 5.2%, and 14.2%, respectively. The overall prevalence of cardiac dysfunction was significantly higher in lymphoma survivors in comparison to controls (20.6% versus 3.9%, *p* < 0.01). In multivariable logistic regression analysis, the odds of having cardiac dysfunction were 6.6-fold (OR: 6.6, 95% CI 2.5–21.1; *p* = 0.01) greater among lymphoma survivors in comparison to matched controls (54). A dose-dependent association with cumulative exposure to anthracycline was shown in comparison to unexposed controls: 1–249 mg/m^2^, OR = 4.7 (95% CI 1.0–17.4), *p* = 0.05; ≥250 mg/m^2^, OR = 7.6 (95% CI 2.7–24.3), *p* < 0.01. The authors emphasized that a global echocardiographic evaluation by 2D-STE echocardiography also should be offered at any time to asymptomatic survivors who had received less than 250 mg/m^2^ of doxorubicin due to the 4.7-fold increase of cardiotoxicity [52]. Despite a demonstrated anthracycline dose-dependent association, an interpatient variability in the risk of developing CHF after anthracycline-containing therapies at any dose was reported, thus underlying the role of genetic factors in determining the final risk [52]. Overall, the quality of the study was intermediate due to selection bias of the exposed cohort and controls selected from a nonexposed population.

##### Nonechocardiographic Imaging and Markers for the Early Detection of LV Dysfunctions: Magnetic Resonance Imaging (MRI), Cardiac Troponins, and Natriuretic Peptide Levels

Machann et al., in their cohort study, evaluated the role of MRI in HL survivors treated with anterior mediastinal RT (cobalt-60, median prescribed dose 40 Gy) between 1978 and 1985. Out of 55 long-term survivors (median 24 years after radiotherapy), 31 (58%) underwent MRI. MRI detected pathologic findings in approximately 70% of survivors. Pathologic findings were reduced LVEF <55% (*n* = 7, 23%), hemodynamically significant valvular dysfunction (*n* = 13, 42%), and ischemic late myocardial enhancement (*n* = 8, 26%); moreover, a perfusion deficit at rest and under stress was found in 61% and 72% of patients, respectively, while the prevalence of any perfusion deficit was 68%. A late increase in gadolinium was recorded in 29% of patients that was unexplainable by other etiologies. The authors concluded that cardiac MRI may be an essential tool in the cardiac imaging of patients treated with radiotherapy, since a single noninvasive examination was able to provide all significant cardiac findings [47]. The quality of the study was overall intermediate, with a good selection of cases and long-term follow-up, and the use of a multiparametric imaging. However, a control group was not present for comparison.

Armenian et coll. evaluated cardiac serologic markers in the above-mentioned cohort study conducted on 155 HL and NHL lymphoma survivors (>5 years), including patients treated with ASCT. Lymphoma survivors had significantly higher B-type natriuretic peptide (BNP) and NT pro-brain natriuretic peptide (NT-proBNP) values in comparison to controls (*p* < 0.01). There was no statistically significant difference in BNP and NT-proBNP values between conventionally treated and ASCT survivors, or in protein ST-2 levels. The sensitivity of blood biomarkers ranged from 6% (ST-2) to 28% (BNP); specificity ranged from 86% (NT-proBNP) to 89% (BNP, ST-2); positive predictive value ranged from 15% (ST-2) to 41% (BNP); and negative predictive value ranged from 78% (ST-2) to 83% (BNP). There was no advancement in the diagnostic accuracy of biomarkers (BNP, NT-proBNP, and ST-2) according to type of cardiac dysfunction (diastolic, systolic abnormal global longitudinal strain), or by specific cutoffs (BNP ≥50 pg/dL, NT-proBNP ≥125 pg/dl). The authors concluded that the diagnostic usefulness of these biomarkers was low in the asymptomatic population [52]. The quality of the study has been previously depicted.

#### 3.3.2. Detection and Monitoring for CHD

Stress tests and myocardial scintigraphy/MRI, computed tomographic coronary angiography (CTA), and coronary artery calcium (CAC) score measurements were used as diagnostic tools.

All included studies concerned HL survivors.

From 2005 to 2007, Andersen et al. performed CAC-score measurements (Agatson and volume scores) in 47 cHL treated with mediastinal radiotherapy from 1980–1988 who had survived ≥15 years after therapy. In this cohort, 57% of patients were treated with anthracycline-containing chemotherapy. Seven patients already had ascertained CHD and had high CAC. Of the residual 40 cHL survivors, eight had a CAC of 0. A total of 27 had a CAC between 1 and 199, and 5 had a CAC score between 200 and 999 [53].

The authors observed that patients with a CAC score >200 often had clinically important CHD; on the other hand, lower CAC scores did not exclude CHD. The quality of the study was intermediate, because despite the good case definition and assessment of the outcomes, the main bias could be represented by the small sample size, the absence of follow-up data after screening of this cohort, and the fact no angiography of coronary arteries (CAG) was done to assess the severity of CHD.

From 2011 to 2012, Daniels LA and al. examined the role of computed tomographic coronary angiography (CTA) in a cohort of 52 HL survivors (median time since HL diagnosis: 21 years) undergoing mediastinal irradiation. Thirty patients out of the total were treated with anthracycline-containing chemotherapy. The median dose of mediastinal RT was 36 Gy (range 24–40 Gy). The prevalence rate of significant CHD on CTA (45 evaluable scans) was 20% (*p* = 0.01, 95% CI 8.3% to 31.7%) Patients with relevant CHD on CTA more often had high CAC scores (75th–100th percentile) than patients who had no severe anomalies on the CTA scan (55% compared with 17%). The quality of the study was intermediate; although the case definition and assessment of the outcomes were good, the main bias could be represented by the small sample size [54].

Heidenreich et al. enrolled 294 cHL outpatients evaluated at a tertiary care cancer center (from 1994 to 1998) after mediastinal irradiation doses ≥35 Gy (from 1964–1994) who had no known CHD [50]. Stress echocardiography and radionuclide perfusion imaging were performed at one stress session. The decision to undergo coronary angiography was a discretionary medical choice. The median follow-up after screening was 6.5 years. They found a 2.7% prevalence of severe, three-vessel, or left main coronary artery disease, and a 7.5% prevalence of coronary stenosis greater than 50% in patients treated with mediastinal irradiation in doses of ≥35 Gy for cHL at a mean of 15 years after RT. The authors concluded that stress testing was able to identify asymptomatic people at high risk of acute myocardial infarction or sudden cardiac death. Conventional risk factors for CHD were infrequent and did not predict coronary stenosis. The authors recommend starting screening 5 years after initial therapy. The quality of the study was overall intermediate, with a good selection of cases and duration of the follow-up after screening. The main concern was the lack of information about concomitant chemotherapy, and in particular about anthracyclines, in this cohort of patients.

In the above-mentioned cohort study by Machann et al., the authors emphasized that in cHL survivors treated with anterior mediastinal RT (cobalt-60, median prescribed dose 40 Gy) between 1978 and 1985, MRI evaluation was a very useful tool for myocardial perfusion assessment for ischemia [47]. The quality of the study has been previously depicted.

#### 3.3.3. Detection and Monitoring for Valvular Disorders

There is a lack of studies planned to show the benefit of close monitoring for valvular disorders in lymphoma survivors. All valvular abnormalities were detected by echocardiography in published studies.

In 1993, Wethal T et al., studied changes in valvular and myocardial function related to therapy with mediastinal (fractional doses of 1.8 or 2.0 Gy 5 days per week, median dose of 40.0 Gy, 43% mantle field, 8% mediastinal field only) and anthracyclines in HL survivors. A total of 116 patients after a median of 10 years from treatment with mediastinal RT underwent an echocardiogram. Between 2005–2007, 51 patients were enrolled in a second echocardiographic study (median 22 years after treatment). Of these patients, 28 (55%) had also received anthracyclines (with a total median adriamycin dose of 320 mg in 27 and 720 mg of epirubicin in 1 patient). The patients were selected on the basis of the presence or absence of moderate valvular regurgitation in 1993. The authors concluded that cHL patients treated with mediastinal RT were at a high risk of developing growing valvular impairment and aortic stenosis 20 years after initial therapy. Treatment regimens containing anthracyclines were associated with LV remodeling. These results underlined the need to extend the cardiological follow-up for these patients, considering the late cardiovascular side effects of radiotherapy and chemotherapy in cHL survivors [55]. The quality of the study was intermediate; despite the good case definition, assessment of the outcomes, and a longitudinal follow-up design, it was limited by the small sample size and the lack of a control group of healthy individuals. The longitudinal follow-up design was able to demonstrate the slow progressive nature of valvular dysfunction in individual patients independent of valvular involvement at the first echocardiographic study.

Bijl JM et al. evaluated a cohort of 82 cHL survivors from 2007 to 2008 (52% men, mean age 47.8 years, 50 patients undergoing mediastinal RT) [56]. Valvular disease was diagnosed by transthoracic echocardiography and compared between HL survivors treated with and without mediastinal RT. Valvular disease predictors were identified by univariate and multivariate logistic regression analysis. During a median follow-up of 13.4 years, 61.2% (*n* = 30) of HL survivors with mediastinal RT had ≥mild valvular disease, compared with 31% of HL survivors without RT (OR 3.51, 95% CI 1.32 to 9.30, *p* = 0.01). The authors concluded that the prevalence of valvular disease in MRT-treated HL survivors was high and increased with time after RT. The authors stressed the utility of long-term periodic screening for valvular disease using transthoracic echocardiography. Study quality was intermediate; despite the good case definition, the evaluation of the results, and a longitudinal follow-up design, it was limited by the small sample size and the lack of a control group of healthy people.

#### 3.3.4. Detection and Monitoring for Pericardial Disease

In the cohort study by Heidenreich et al., 254 asymptomatic HL survivors treated with RT (at least 35 Gy) and a LV block were screened for pericarditis using echocardiographic evaluation [57]. A total of 21% of patients had a thickened pericardium and 3% small pericardial effusions, while none of the patients had signs suggestive of constricting pericarditis. The quality of the study was intermediate, and the average follow-up after echocardiography was less than 4 years in this study. Time from irradiation to examination varied from 2 to 33 years [57].

## 4. Discussion

Survival of patients treated for lymphoma has improved greatly in the last decades thanks to the development of multiagent chemotherapy and more accurate radiotherapy techniques. The improvement of survival has led to an ever-increasing number of long-term lymphoma survivors and consequent long-term toxicities. The standard therapy for the majority of patients diagnosed with cHL and DLBCL includes anthracycline-containing regimes; mediastinal irradiation is also indicated for a large portion of mainly cHL patients. Both these approaches have been shown to strongly increase the risk of cardiovascular events in long-term survivors [1,3]. Although the reported cure rates in patients affected by lymphoma are currently high, reaching a 10-year survival rate of more than 80% for cHL, morbidity and mortality from cardiovascular causes in these patients are still a clinical problem [1,3,42].

Although cardiotoxicity is the best-known and studied among long-term toxicities over the years, the optimal follow-up for these at-risk patients is currently unclear. The multidisciplinary team of FIL researchers, comprising two cardio-oncologists, one radiotherapist, four onco-hematologists and two methodologists, aimed to clarify, through a systematic review of the literature with the PRISMA methodology, evidence about incidence, comparison between therapies (including liposomal anthracyclines) and radiotherapy with modern techniques, and the better monitoring of long-term cHL and DLBCL survivors. The research focused on patients treated in adulthood and with first- or second-line antineoplastic therapies, including ASCT.

The authors limited the systematic review to the two histotypes that prevalently constitute the population of lymphoma survivors: classical Hodgkin lymphoma and diffuse large B-cell lymphoma. All the remaining histotypes belonging to NHL were deliberately omitted in order to have a selected population. In some cases the treatments were different, but the majority of the studies included patients treated with ABVD or R-CHOP.

FIL researchers confirmed that long-term cHL survivors presented a strongly increased risk of developing cardiovascular events when compared to the general population, with a risk of MI and CHF of 3.6 and 4.9, respectively [42]. The cumulative risk over time for CVD in this population was 3–5-fold more compared to the age-matched general population [42]; in this study, age-, sex-, and calendar-period-specific incidence rates were used for comparison. In a large cohort study on long-term cHL survivors treated with radiotherapy, the risk of CHD increased over time during follow-up, reaching 9% at 10 years, 15% at 20 years, 26% at 30 years, and 30% at 40 years follow-up [38]. The median age at the first CV event was 52–53 years for ischemic heart disease, cardiomyopathy, and valvular disease [34]. At a median follow-up duration of 9 years, cHL survivors treated with both chemotherapy and radiotherapy presented a 19% risk of ischemic heart disease, 12% of CHF, 16% of arrhythmias, and 11% of valvular disease [35]. Mediastinal RT increased the risk of myocardial infarction, angina pectoris, CHF, and valvular disorders at 2–7-fold, while anthracyclines increased the risk for CHF at 2.82-fold [42]. For doses above 30 Gy, a significant increase of valvular heart disease was shown over time [41]. Finally, using the tool of late morbidity survey and at a median follow-up of 22 years, Matasar et al. described CV morbidity in 54.6% of cHL survivors, of whom 17.3% presented grade 3–4 morbidities [40].

In the included study regarding DLBCL survivors, at an 8-year follow-up, any doxorubicine use was associated with a 29% increased risk of CHF. This risk also correlated with other CV risk factors, such as age, prior heart disease, diabetes, and hypertension. Hypertension demonstrated a synergistic negative effect with doxorubicin, with a hazard ratio of 1.8 [58]. Anthracycline-induced CHF exhibited a dose-response relationship [43].

Armenian et al. evaluated the risk of CVD in the population of lymphoma survivors after ASCT. Compared with the sex- and age-matched general population, the risk of CHF for ASCT survivors was 4.5-fold, with an increased risk with increased age at transplant [39].

Several strategies to decrease CVD incidence in lymphoma long-term survivors have been proposed. The use of less cardiotoxic analogs such as liposomal doxorubicin is one of these; another strategy is a prolonged infusion duration. The systematic review did not identify any long-term studies involving lymphoma survivors treated with liposomal anthracyclines, due to the unavailability of studies with long-term follow-up, although clinical benefit was documented in the years immediately following treatment [59].

In recent decades, using dosimetry parameters as surrogate endpoints for the risk of late toxicity, the introduction of smaller RT field sizes has been related to a reduction of the radiation exposure to organs at risk (heart, lung, breast, and thyroid) and these dosimetry improvements are likely to translate into lower rates of RT-induced toxicity. Therefore, with the adoption of IFRT and, more recently, of INRT or ISRT, we will witness in the near future a reduction of radiotherapy-related cardiac toxicities in Hodgkin and non-Hodgkin long-survival patients [24,60]. Another strategy of minimizing heart radiation exposure is the employment of highly conformal RT techniques such as IMRT or volumetric modulated arc therapy (VMAT) eventually associated with the implementation of breath-hold techniques [61,62,63,64]. To understand whether dose reductions and technological changes had led to a reduced impact on CVD, only three studies were eligible for the systematic review and regarded the population of cHL survivors [35,36,37,38,39,40,41,42,43,44,45,58]. A trend of reduced CV events and all-cause mortality was reported in patients treated after 1997 in a large retrospective registry [44]. According to the available data and when looking for specific volumetric and dosimetry data and the volume of the irradiated cardiac structures, no conclusion could be formulated on the specific PICO. The authors also evaluated dosimetry studies that could be able to predict the risk of CVD. These studies are available prevalently in the population of cHL. Van Nimwegen et al. calculated the mean heart doses and mean left ventricular doses (MLVD) of radiotherapy performed through parallel-opposed fields, and demonstrated in 2617 cHL a linear correlation between the radiation dose and the risk for CHD that increased by 7.4% for each Gy [38]. They also calculated in another case-control study the 25-year risk of CHF following MLVD of 0–15 Gy 11.2%, 16–20 Gy 15.9%, and ≥21 Gy 32.9% in patients also treated with anthracyclines [37]. Similar results were calculated retrospectively in large clinical trials by the EORTC [35]. Finally, although not largely available in clinical practice, proton therapy (PT) significantly decreased all dose/volume metrics of the OARs, suggesting that IMPT can be a technique with a high potential to reduce cardiac toxicities in long survival lymphoma patients [65]. Moreover, for a modern radiotherapy and a correct dosimetry approach, it is now strongly suggested to contour not only the heart “in toto”, but also all the main cardiac substructures according to the published atlas [66,67]. This is in order to record the radiation dose delivered to each cardiac substructure, but mainly to optimize the radiation dose distribution so as to lower as much as possible the dose to the identified and contoured cardiac structures [68]. For this reason, currently lymphoma patients are treated with a radiation dose and volume based on the modern concepts delivered with sophisticated RT techniques, and to a lesser extent with proton therapy (PT). In this context, after individualizing and optimizing the radiation dose to the main cardiac substructures, data on the estimation doses to the heart using smaller fields such as INRT or ISRT, delivered with VMAT or intensity-modulated spot-scanned PT, demonstrated a reduced estimated risk of CVD in comparison with extensive techniques used in the past for patients with early-stage, mediastinal cHL [68].

FIL researchers therefore wanted to collect evidence about the optimal monitoring of the described forms of CVD, such as long-term sequelae of cHL and treatment. The study of cardiac serum markers; e.g., TnI and NT-pro-BNP, showed no evidence of utility in the long-term follow-up of these patients [39] and therefore are not indicated. Regarding the early identification of LV dysfunction, an accurate monitoring can be obtained by two-dimensional speckle tracking echocardiography (2D-STE) [47,51]; 2D-STE-derived strain and strain rate parameters (global longitudinal strain (GLS), circumferential strain of subendocardial layer, and global circumferential strain (GCS)) [46] can identify changes in myocardial functions before alterations in LVEF occur, and can predict a future decrease in EF to less than 50%, or of greater than 10%, indicative of cardiotoxicity [39,48,69]. Three-dimensional echocardiography-derived LVEF has an excellent correlation with cardiac MRI, which also allows the simultaneous evaluation of multiple LV function parameters in the follow-up of cHL survivors [47]. The routine use of cardiac MRI in follow-up is not yet feasible. However, when available, it is a very useful tool for evaluating changes in ventricular volumes and ejection fraction, particularly in patients with poor quality echocardiographic images, or if evaluation of myocardial perfusion for ischemia is also planned. Cardiac MRI is an optimal test for the global evaluation of pericardial diseases [70].

FIL researchers therefore suggest a comprehensive evaluation of these parameters (LVEF, GLS, GCS, and MRI parameters).

Currently, there is no score available to predict the individual CV risk of each long-term cHL or DLBCL survivor, or of lymphoma survivors in general, and there are no univocal indications about the timing and method of monitoring cardiotoxicity. Following the evidence that emerged from the systematic review, FIL researchers therefore suggest that survivors with the following myocardial risk profile should be considered as high-risk for developing CHF: doxorubicin dose >250 mg/m^2^ and mediastinal radiotherapy >30 Gy [10,33,35,54,56,61]; additional risk factors to be considered are: age at treatment <25 years [8] and assessment at age >60 years, or with generic CV risk factors [10]. The remaining patients could be considered at standard risk for CVD. Although no studies are available indicating the optimal timing for early detection and monitoring of asymptomatic LV dysfunction, FIL researchers agree with the guidelines for performing an ECG plus 2D-STE echocardiographic study at 5–10 year intervals in standard-risk patients [16], while they suggest that this interval should be less than 5 years in patients at high CV risk due to the presence of the factors listed above, and then every 1–3 years. The authors also agree that a close control of CV risk factors and the promotion of healthy lifestyles should be stressed [71].

The clinical presentation of CHD consists of angina pectoris, myocardial infarction, and sudden death, and could be asymptomatic in 3–4% of cases. The best exam to detect asymptomatic CHD is still being debated [16,71]. Invasive examinations, such as the gold standard coronary angiography, cannot be offered to all asymptomatic patients. Noninvasive, stress-induced ischemia detection by echocardiography, ECG, and scintigraphy has been correlated with coronary angiography with a good specificity. Other diagnostic tools such as CT-angiography and the coronary artery calcium (CAC) score showed promising results in small cohorts of cHL survivors [53]. Incidental coronary calcium in thoracic CT (during staging, RT planning, and subsequent follow-up) should be reported and quantified according to recommendations from the Society of Cardiovascular Computed Tomography [54]. Coronary artery calcification is incrementally associated with worse CV outcomes, involving prescription of preventive therapies [72].

In their review, van Leeuwen-Segarceanu et al. exhaustively summarized the monitoring of CHD in cHL survivors treated with mediastinal radiotherapy. According to the reviewed available literature, the authors identified some risk categories of patients to whom a tailored follow-up of the CHD should be given: (i) patients with more than one concomitant traditional cardiovascular risk factor; (ii) patients treated with more than 15 Gy to the heart; and (iii) patients treated with both chemotherapy and radiotherapy. The authors concluded by screening mainly these categories of survivors of cHL for CHD, starting 5 years after mediastinal RT in patients older than 45 years, and after 10 years in younger patients. The screening should be started earlier, when many cardiovascular risk factors coexist, and in the presence of any referable symptoms [73].

With regard to early detection and monitoring of CHD, FIL researchers suggest, on the basis of the available evidence, an individual risk assessment of this complication, starting from the 10th year after mediastinal radiotherapy for patients aged <45 years [50]. For asymptomatic survivors at risk for RT treatment and CV risk factors, screening with noninvasive methods such as ECG, stress-echo, or CAC measuring may be considered at intervals calculated on the basis of individual risk [73]. The CAC measurement at a 5-year interval for cHL survivors with concomitant cardiovascular risk factors could be a valid option [73]. A strict control of cardiovascular risk factors is always recommended.

The monitoring of asymptomatic valvular heart and pericardial diseases should also be included in the follow-up assessment. Primarily, in the first decade, RT produces leaflet retraction of valvular tissue with related regurgitation, then in the second decade and later, RT induces calcification, thickening, and fibrosis of valvular tissue with progressive stenosis, regardless of patient age and traditional risk factors [74]. There is a lack of planned studies to show the benefit of a close monitoring for valvular disorders in asymptomatic cHL survivors treated with mediastinal radiotherapy. As the risk for valvular disorders starts mainly after 10 years from mediastinal radiotherapy and the election examination is represented by echocardiography, the monitoring of this disorder should be included within the normal cardiac evaluation.

Although late toxicities related to mediastinal RT will be decreasing in the coming years, we still see patients treated with the old RT technologies and with anthracyclines. For each of these long-term survivors, the individual risk of CHF and CHD needs to be carefully assessed in order to plan appropriate detection and monitoring in the asymptomatic phase of the disease. Those patients should be informed and closely monitored [71].

## 5. Conclusions

In conclusion, late CV events still represent a frequent cause of morbidity and mortality for cHL and DLBCL long-term survivors. An early detection, treatment, and monitoring of EF reduction and CHD could ameliorate the prognosis and quality of life of our patients. In parallel, the tertiary prevention of these events plays a fundamental role, and could be obtained with the correction of CV risk factors and of unhealthy lifestyle factors. Many indications for the early detection and monitoring of secondary CDV derive from retrospective or uncontrolled studies; thus, data to support definitive recommendations on various tests and their frequency during follow-up of long-term lymphoma survivors still need to be implemented through ad hoc trials. Our goal was, through a systematic review of all the literature with a standardized approach (PRISMA), to provide information on specific questions such as incidence, risk factors, preferable cardiovascular monitoring over the years, and prevention of cardiotoxicity in the future. The amount of material is enormous, and being able to summarize it in a review that serves as a starting point for future developments seemed useful to us. Now, physicians can benefit from a unique and updated document on incidence, risk factors, early detection, and tertiary prevention of the cardiovascular late effects of chemotherapy in HL and DLBCL survivors. Further studies are also needed to detect a predictive score of cardiotoxicity, and to design a personalized surveillance that could translate into a modification of patient outcome. A multidisciplinary collaboration between hematologists, radiotherapists, and cardiologists constitutes the basis for future clinical research projects aimed at bridging existing gaps and answering unresolved clinical questions.

## Figures and Tables

**Table 1:** Clinical questions and PICOs considered by the review PICO, population, intervention, control, and outcome. cHL, classical Hodgkin lymphoma; DLBCL, diffuse large B-cell lymphoma; ASCT, autologous stem cell transplant; RCTs: randomized controlled trials; LVEF: left ventricular ejection fraction; MRI: magnetic resonance imaging.

What is the incidence of CVD in long-term survivors of cHL or DLBCL after first-line treatment?	P: patient population in long-term survivors of cHL or DLBCL (>5 years free of disease and treatment), with age >18 years at diagnosis;
	I: chemotherapy or chemotherapy + standard dose radiotherapy;
	C1: homogeneous population for age and sex untreated (control group);
	C2: long-term survivors of cHL or DLBCL (>5 years free of disease and treatment) treated with different therapeutic regimens, without anthracyclines;
	O: diagnosis of cardiotoxicity of any degree;
	S: RCTs, retrospective registry studies, (controlled) cohort studies, and any reviews of such studies.
What is the incidence of CVD in long-term survivors of cHL or DLBCL after second-line treatment (including ASCT)?	P: patient population in long-term survivors of cHL or DLBCL (>5 years free of disease and treatment), with age >18 years at diagnosis;
	I: second-line chemotherapy and autologous transplantation, radiotherapy;
	C1: homogeneous population for age and sex untreated (control group);
	C2: long-term survivors of cHL or DLBCL (>5 years free of disease and treatment) treated with other chemotherapy/radiation treatment regimens;
	O: diagnosis of cardiotoxicity of any degree;
	S: RCTs, retrospective registry studies, (controlled) cohort studies, and any reviews of such studies.
Has the incidence of cardiotoxicity in long-surviving patients with cHL or DLBCL changed with the introduction of modern radiotherapy and liposomal doxorubicin after first-line treatment?	P: patient population in long-term survivors of cHL or DLBCL (>5 years free of disease and treatment), with age >18 years at diagnosis;
	I1: new chemotherapy approaches;
	I2: new radiotherapy approaches;
	C1: previous chemotherapy/radiotherapy regimens
	O: diagnosis of cardiotoxicity of any degree;
	S: RCTs, retrospective registry studies, (controlled) cohort studies, and any reviews of such studies.
Has the incidence of cardiotoxicity in long-surviving patients with cHL or DLBCL changed with the introduction of modern radiotherapy after second-line treatment (including ASCT)?	P: patient population in long-term survivors of cHL or DLBCL (>5 years free of disease and treatment), with age >18 years at diagnosis;
	I1: new radiotherapy approaches;
	C: previous radiotherapy regimens
	O: diagnosis of cardiotoxicity of any degree;
	S: RCTs, retrospective registry studies, (controlled) cohort studies, and any reviews of such studies.
How to perform an optimal cardio-oncological follow-up in long-term survivors of cHL or DLBCL after first-line treatment? Which parameters to evaluate and how often?	P: patient population in long-term survivors of cHL or DLBCL (>5 years free of disease and treatment), with age >18 years at diagnosis;
	I1: multiparametric assessment that includes specialist cardiology visit and calculation of LVEF with conventional echocardiography, strain-rate calculation, monitoring with biomarkers, monitoring with MRI, monitoring with MUGA-scan;
	I2: timing validation of the cardiological assessment during the follow-up;
	C: specialist cardiology visits and calculation of LVEF with conventional echocardiography;
	O: diagnosis of cardiotoxicity of any degree;
	S: RCTs, retrospective registry studies, (controlled) cohort studies, and any reviews of such studies.
How to perform an optimal cardio-oncological follow-up in long-term survivors of cHL or DLBCL after second-line treatments (including ASCT)? Which parameters to evaluate and how often?	P: patient population in long-term survivors of cHL or DLBCL (>5 years free from disease and treatments), with age >18 years at diagnosis, already treated in the first line with chemotherapy/radiotherapy according to standard schedules and dosages;
	I1: multiparametric evaluation that includes specialist cardiology visit and calculation of LVEF with conventional echocardiography, strain-rate calculation, monitoring with biomarkers, monitoring with MRI, monitoring with MUGA-scan;
	I2: validation of the timing of the cardiological assessment during the follow-up;
	C: specialist cardiology visits and calculation of LVEF with conventional echocardiography;
	O: diagnosis of cardiotoxicity of any degree;
	S: RCTs, retrospective registry studies, (controlled) cohort studies, and any reviews of such studies

**Table 2:** Summary of findings.

Summary of Findings				
**PICO 1**				
**Study**	**Study Design and Sample Size**	**Intervention and Comparison**	**Primary Outcomes**	**Secondary Outcomes**
Holtzman A.L. 2019 [34]	Retrospective cohort study (365 cHL patients)	Primary RT	9% of CHD incidence at 10 years, 15% at 20 years, 26% at 30 years, 30% at 40 years of follow-up	-
Maraldo M.V. 2015 [35]	Retrospective cohort study (6030 cHL patients)	Primary CT with anthracycline and vinca-alkaloid; RT	19% of CHD, 12% CHF, 16% arrhythmia, 11% VHD incidence	The mean heart radiation dose per 1 Gy increase and the dose of anthracylines were significant predictors of CHD
Clavert A. 2016 [36]	Retrospective cohort study (110 cHL e NHL)	Allotransplant	47% of CVD incidence at 10 years (95% CI 35–59), and main presentations were heart failure (14%) and arterial hypertension (7%)	-
van Nimwegen F.A. 2015 [8]	Retrospective cohort study (2524 cHL patients)	RT only, or RT and CT (with or without anthracycline), or CT only (with or without anthracycline)	The 40-year cumulative incidence of CVD was 50% (95% CI, 47–52%)	-
van Nimwegen F.A. 2017 [37]	Case-control study (2617 cHL patients)	RT; anthracycline-containing CT; without anthracycline-containing CT	25-year cumulative risks of heart failure following mean left ventricular doses of 0–15 Gy, 16–20 Gy, and ≥21 Gy were 4.4%, 6.2%, and 13.3%, respectively, in patients treated without anthracycline-containing CT; and 11.2%, 15.9%, and 32.9%, respectively, in patients treated with anthracyclines	-
van Nimwegen F.A. 2016 [38]	Nested case-control study (2617 cHL)	RT; anthracycline-containing CT; without anthracycline-containing CT	2.5-fold risk of CHD for patients receiving a MHD of 20 Gy from mediastinal radiotherapy	Risk of CHD increased linearly with increasing MHD (excess relative risk [ERR]) per Gray, 7.4%; 95% CI, 3.3% to 14.8%)
Galper S.L. 2011 [7]	Retrospective cohort study (1279 patients)	Mediastinal RT or mediastinal RT and CT	5-, 10-, 15-, and 20-year cumulative incidence rates of cardiac events were 2.2%, 4.5%, 9.6%, 16%	-
Armenian S.H. 2011 [39]	Retrospective cohort study (NHL 598, cHL 284 patients) Case-control study (88 patients)	Hematopoietic cell transplantation or conventional therapy	The incidence of CHF for HCT survivors was increased 4.5-fold compared with the controls	The cumulative incidence of CHF was 4,8% at 5 years and 9.1% at 15 years after HCT
Stone C.R. 2019 [33]	Systematic review and meta-analysis (22 studies, total of 32,438 patients)	Treatment for lymphoma	Relative to the general population, lymphoma survivors had statistically significant 2- to 3-fold increases in the risk for CVD	-
Matasar M.J. 2015 [40]	Retrospective cohort study (746 cHL patients)	First-line therapy for HL	Mortality: 30.4% of patients had died, 47.1% from HL, and 52.9% from other causes, including second primary malignancies (n2) and CVD (27)	Cardiovascular morbidity (54.6%)
Cutter D.J. 2015 [41]	Case-control study (1852 cHL patients)	RT	VHD incidence: 89 case patients with VHD were identified (66 severe or life-threatening)	For doses above 30 Gy the percentage growth in VHD rate per Gy increases progressively with increasing dose
Aleman B.M. 2007 [42]	Retrospective cohort study (1474 cHL patients)	RT only (27.5%), CT only (4.8%), RT + CT-anthracylines (29.5%), RT + CT no anthracylines (37.9%), unknown (8.2%)	The 25-year cumulative incidence of CHF after mediastinal RT and anthracyclines in competing risk analyses was 7.9%	-
Baech J. 2018 [43]	Retrospective Cohort study (2440 NHL patients)	Anthracycline-containing CT (R-CHOP or R-CHOEP); CT without anthracyclines	Patients treated with 3–5 cycles of R-CHOP/CHOEP had risks of CVD at 1, 5, and 8 years of 2.5%, 10.5%, and 17.2%, respectively	-
**PICO 2**				
**Study (Reference)**	**Study Design and Sample size**	**Intervention and Comparison**	**Primary Outcomes**	**Secondary Outcomes**
Patel C.G. 2017 [44]	Retrospective cohort study (1541 cHL patients)	1968–1982: RT field TNI 11.6%, mantle/para-aortic 73.4%; mantle 9.2%, inverted Y/pelvis 4.1%, IFRT 1.6%; 1983–1992: RT fields TNI 1,4 mantle/paraaortic 59.4%; mantle 33.1%, inverted Y/pelvis 3.2%, IFRT 3.0%; 1993–2007: RT fields TNI 0%, mantle/para-aortic 5.6%, mantle 40.9%, inverted Y/pelvis 3.4%, IFRT 42.2%, 0	15-years OS rates were 78%, 85%, and 88% (*p* < 0.01) according treatment periods	-
Fu J. 2017 [45]	Systematic review (articles from 1990–2016)		No studies fulfilled all review criteria (assessing the risk of cardiac toxicity after contemporary treatment for HL)	-
Matasar M.J. 2015 [40]	Retrospective cohort study (746 cHL patients)	CT containing anthracycline; RT in doses from 20 to 36 Gy	Patients treated with less than 35 Gy did not have lower rates od CHD	-
**PICO 3**				
**Study**	**Study Design and Sample Size**	**Intervention and Comparison**	**Primary Outcomes**	**Secondary Outcomes**
Kang Y. 2018 [46]	Cohort study (45 NHL patients)	Echocardiographic imaging; multilayer speckle tracking echocardiography	Compared with controls, patients had no different conventional parameters of systolic and diastolic function, but significantly lower GCS and GLS, significant reduction of circumferential strain (CS) of subendocardial layer, transmural CS gradient, and longitudinal strain of all three layers	In contrast, the two groups did not differ in transmural longitudinal strain gradient and radial strains
Machann W. 2011 [47]	Cohort study (31 cHL patients)	MRI in patients treated with mediastinal RT	Pathologic findings were reduced LVEF (<55%) in 23%of patients, hemodynamically relevant VHD in 42%, late myocardial enhancement in 29%, and any perfusion deficit in 68% of patients	-
Nolan M.T. 2016 [48]	Systematic review (21 studies, total of 1659 patients)	10 studies used transthoracic echocardiography (TTE), 8 used radionuclide ventriculography (RNV), 2 used TTE and RNV, and 1 used cardiac MRI	LVEF presented limitations for the identification of mild or subclinical LV systolic dysfunction	2D speckle tracking strain, showed more accuracy and reproducibility in diagnosing subclinical systolic dysfunction
Tsai H.R. 2010 [49]	Cohort study (47 cHL patients)	Left ventricular function assessed by 2D speckle tracking echocardiography	The global longitudinal strain was reduced in patients receiving anthracycline with mediastinal RT compared to the other group receiving mediastinal RT alone or combined RT and regimens without anthracyclines (16.1 1.9% vs. 17.5 1.7%, respectively, *p* < 0.05). Both patient groups had reduced strain compared to the healthy controls (20.4 1.7%, both *p* < 0.001). The circumferential strain was also reduced in the treatment groups (18.3 3.2% and 17.8 3.6% vs. 22.5 2.1%, both *p* < 0.001)	The LV ejection fraction did not differ between the patient groups but was reduced compared to that of the controls
Heidenreich P.A. 2007 [50]	Cohort study (294 cHL patients)	Stress echocardiography (97% exercise, 3% dobutamina) in all patients; in 274 patients, radionuclide perfusion imaging	A 2.7% prevalence of severe, three-vessel, or left main coronary artery disease, and a 7.5% prevalence of coronary stenosis greater than 50% in patients treated with mediastinal RT in doses of ≥35 Gy for HL at a mean of 15 years following irradiation	-
Murbreach K. 2015 [51]	Prospective cross-sectional study (274 cHL patients)	Echocardiographic imaging; LVEF assessed by Simpson’s biplane rule	In the multivariable analysis, only doxorubicin ≥ 300 mg/m^2^ (OR, 3.3; 95% CI, 1.2 to 8.9; *p* = 0.02) and cardiac-RT more than 30 Gy (OR, 4.3; 95% CI, 1.7 to 11.4; *p* = 0.003) remained significantly associated with LV systolic dysfunction	-
Armenian S.H. 2018 [52]	Cohort study (155 cHL and NHL patients)	Monodimensional/2D echocardiographic imaging; 2D speckle tracking echocardiography	At a median follow-up of 9.4 years from diagnosis, one in five (20.6%) lymphoma survivors had cardiac dysfunction; The prevalence of reduced LVEF, diastolic dysfunction, and abnormal GLS was 8.4%, 5.2%, and 14.2%, respectively	A dose-dependent association with cumulative exposure to anthracycline was shown in comparison to not exposed controls: 1–249 mg/m^2^, OR = 4.7 (95% CI 1.0–17.4), *p* = 0.05; ≥250 mg/m^2^, OR = 7.6 (95% CI 2.7–24.3), *p* < 0.01
Andersen R. 2010 [53]	Cohort study (47 cHL patients)	CAC-score CTA	The prevalence rate of significant CHD on CTA was 20% (*n* = 9, *p* = 0.01, 95% confidence interval 8.3% to 31.7%)	Patients with a CAC-score >200 often have clinically important CAD (55% compared with 17%)
Daniels L.A. 2014 [54]	Cohort study (52 cHL patients)	CAC-score CTA	Patients with relevant CHD on CTA more often had high CAC scores (75th–100th percentile) than patients who had no severe anomalies on the CTA scan (55% compared with 17%)	-
Wethal T. 2009 [55]	Cohort study (116 cHL patients)	2D echocardiographic imaging	cHL patients treated with mediastinal RT were at a high risk of developing growing valvular impairment and aortic stenosis 20 years after initial therapy	-
Bijl J.M. 2016 [56]	Cross-sectional study (82 cHL patients)	2D echocardiographic imaging	≥mild valvular disease was present in 61.2% of HL survivors with mediastinal RT (*n* = 30), compared with 31.0% of HL survivors without MRT (*n* = 9; odds ratio (OR) 3.51, 95% CI 1.32 to 9.30, *p* = 0.01)	-
Heidenreich P.A. 2003 [57]	Cohort study (254 cHL patients)	Monodimensional/2D echocardiographic imaging	21% had a thickened pericardium, and small pericardial effusions were present in 3%, no patients had wall-motion abnormalities or Doppler findings suggestive of constrictive pericarditis	-

RT, radiotherapy; CT, chemotherapy; CVD, cardiovascular disease; CHD, coronary heart disease; CHF, congestive heart failure; VHD, valvular heart disease; LVEF, left ventricular ejection function; MHD, mean heart dose; CAC-score, coronary artery calcium score; CTA, coronary angiography; MRI, magnetic resonance imaging.

## Data Availability

No new data were created or analyzed in this study. Data sharing is not applicable to this article.

## Supplementary Materials

The following are available online at https://www.mdpi.com/article/10.3390/cancers14010061/s1, Table S1: Search strategies, Table S2: Full text eligibility and data extraction, Table S3: Risk of bias.
