# Supplementary material for: Late Cardiological Sequelae and Long-Term Monitoring in Classical Hodgkin Lymphoma and Diffuse Large B-Cell Lymphoma Survivors: A Systematic Review by the Fondazione Italiana Linfomi
Source: Cancers (Basel). 2021 Dec 23;14(1):61. doi: 10.3390/cancers14010061 (PMC8750391; doi:10.3390/cancers14010061)
Supplement: Supplementary file 1 [file cancers-14-00061-s001.zip › cancers-1403872-Table S1. Search strategies.pdf]

Table S1. Search strategies.

**Example of search strategy used in MedLine and adapted to search the other databases.**
• PICO cardiotoxicity incidence<br><br>MedLine,Embase, and Cochrane databases up to November 2019<br><br>((((((((("hodgkin disease" OR hodgkin disease[mh] OR Lymphoma, Large B-Cell, Diffuse[MH] OR DLBCL[TIAB] OR "diffuse large B cell lymphoma" OR "HODGKIN LYMPHOMA")) AND ("surviv*" OR "long term survivor" OR "survivorship" OR cancer survivors[Mesh])) AND (((((((((((((((((cardiovascular disease) OR "Cardiovascular Diseases"[Mesh]) OR valvular disease) OR diastolic dysfunction) OR systolic dysfunction) OR left ventricular dysfunction) OR heart disease) OR "Heart Diseases"[Mesh]) OR "late cardiotoxicity") OR "cardiotoxicity"[Mesh]) OR cardiomyopathy) OR "Cardiomyopathies"[Mesh]) OR cardiac mortality OR cardiotoxicity OR "cardiovascular toxicity") OR echocardiography) OR "Echocardiography"[Mesh]) OR heart failure) OR "Heart Failure"[Mesh])) AND ((((("Consolidation Chemotherapy"[Mesh] OR "Induction Chemotherapy"[Mesh]) OR "Radiotherapy" OR "Radiotherapy"[Mesh] OR "conventional chemotherapy" OR "chemotherap*" OR "treatment" OR "ABVD" OR "doxorubicin bleomycin vinblastine dacarbazine" OR "COMP" OR "RCOMP" OR "cyclophosphamide doxorubicin vincristine prednisone" OR "RCHOP" OR "CHOP" OR "rituximab cyclophosphamide doxorubicin vincristine prednisone" OR "high dose chemotherapy" OR "autologous stem cell transplant" OR "transplantation" OR "transplant" OR "haematopoietic stem cell graft" OR "brentuximab" OR "Antineoplastic Agents"[MH] OR "Antineoplastic Protocols"[Mesh] OR "Chemoradiotherapy"[Mesh] OR "Chemotherapy, Adjuvant"[Mesh] OR "Antineoplastic Combined Chemotherapy Protocols"[Mesh]))))))))))))
• PICO therapy comparison<br><br>MedLine,Embase, and Cochrane databasesup to November 2019<br><br>((((((cardiovascular disease) OR "Cardiovascular Diseases"[Mesh]) OR valvular disease) OR diastolic dysfunction) OR systolic dysfunction) OR left ventricular dysfunction) OR heart disease) OR "Heart Diseases"[Mesh]) OR "late cardiotoxicity") OR "cardiovascular toxicity") OR "cardiotoxicity"[Mesh]) OR cardiomyopathy) OR "Cardiomyopathies"[Mesh]) OR cardiac mortality) OR echocardiography) OR "Echocardiography"[Mesh]) OR heart failure) OR "Heart Failure"[Mesh])))) AND ((("surviv*" OR "long term survivor" OR "survivorship" OR cancer survivors[Mesh]))))) AND (((("hodgkin disease" OR hodgkin disease[mh] OR Lymphoma, Large B-Cell, Diffuse[MH] OR DLBCL[TIAB] OR "diffuse large B cell lymphoma" OR "HODGKIN LYMPHOMA"))))

- PICO cardiologic follow up

MedLine, Embase, and Cochrane databases up to November 2019

((((("hodgkin disease" OR hodgkin disease[mh] OR Lymphoma, Large B-Cell, Diffuse[MH] OR DLBCL[TIAB] OR "diffuse large B cell lymphoma" OR "HODGKIN LYMPHOMA"))))) AND (((((((global longitudinal strain) OR ((((("muga scan") OR "cardiac magnetic resonance") OR cardiotoxicity monitoring) OR troponin))))))))
